# Supplementary material for: An open state of a voltage-gated sodium channel involving a π-helix and conserved pore-facing asparagine
Source: Biophys J. 2021 Dec 8;121(1):11–22. doi: 10.1016/j.bpj.2021.12.010 (PMC8758419; doi:10.1016/j.bpj.2021.12.010)
Supplement: Document S1. Figures S1–S18 [file mmc1.pdf]

Biophysical Journal, Volume *121*

Supplemental information

An open state of a voltage-gated sodium channel involving a π-helix and conserved pore-facing asparagine

Koushik Choudhury, Marina A. Kasimova, Sarah McComas, Rebecca J. Howard, and Lucie Delemotte

Supplemental Information

An open state of a voltage-gated sodium channel involving a π-helix and conserved pore-facing Asparagine

Koushik Choudhury[1], Marina A. Kasimova[1], Sarah McComas[2], Rebecca J Howard[2], Lucie Delemotte[1]

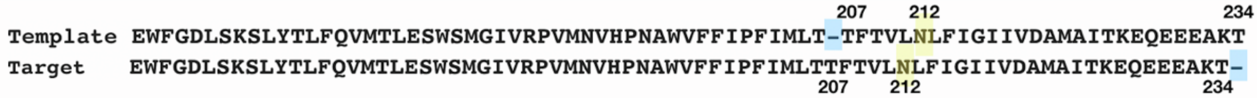

Figure S1: Sequence alignment of the S6 helix of NavMs (UNIPORT ID A0L5S) used as an input to Modeller to build the pi-model. The gap is highlighted in blue and the conserved Asparagine in yellow.

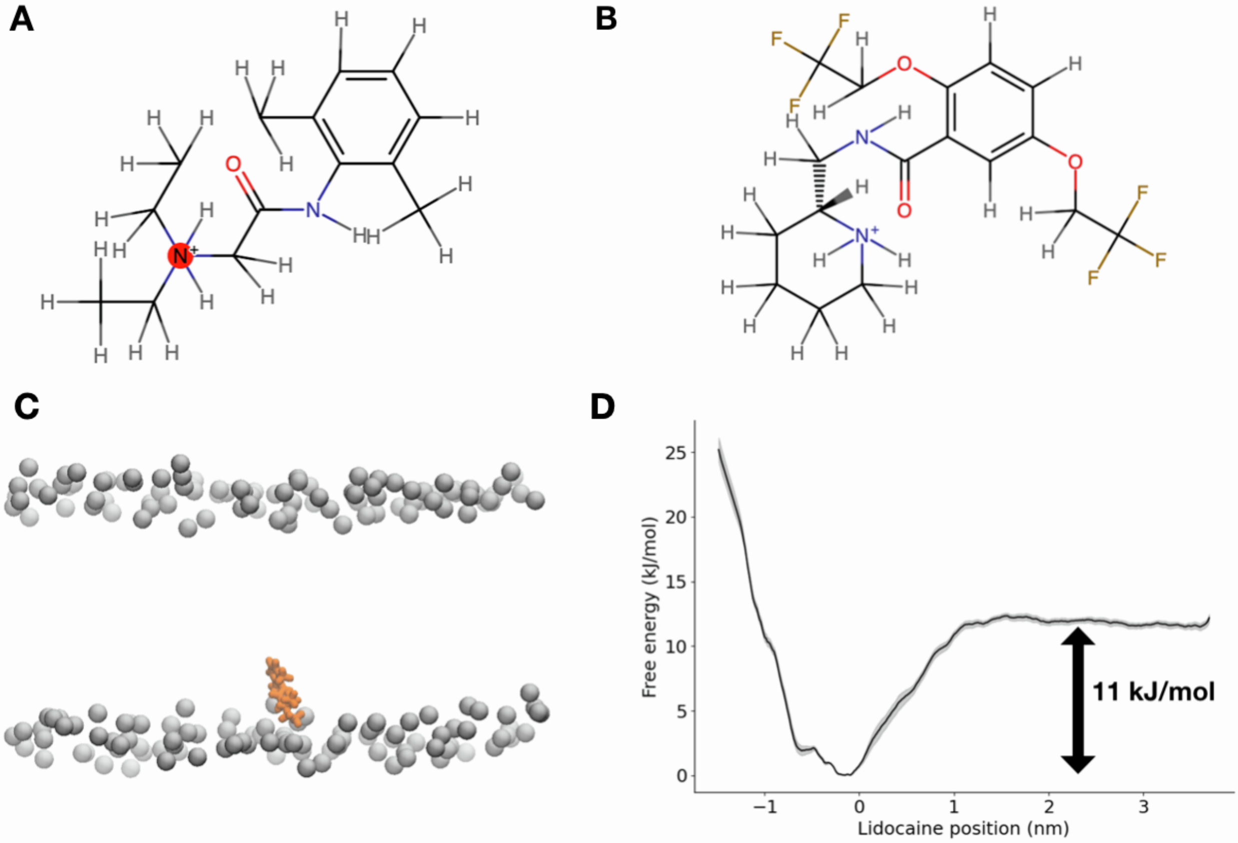

Figure S2. A. Molecular structure of lidocaine and **B.** flecainide. **C.** Lidocaine localization in a POPC membrane. The drug is represented as orange sticks, while the phosphorus atoms of the lipids are shown as grey spheres. **D.** The lidocaine parameters were checked by calculating the free energy for water to membrane transition along the membrane normal, considering one half of the membrane. The free energy difference (indicated by the double headed arrow) for water to membrane transition is around -11 kJ/mol.

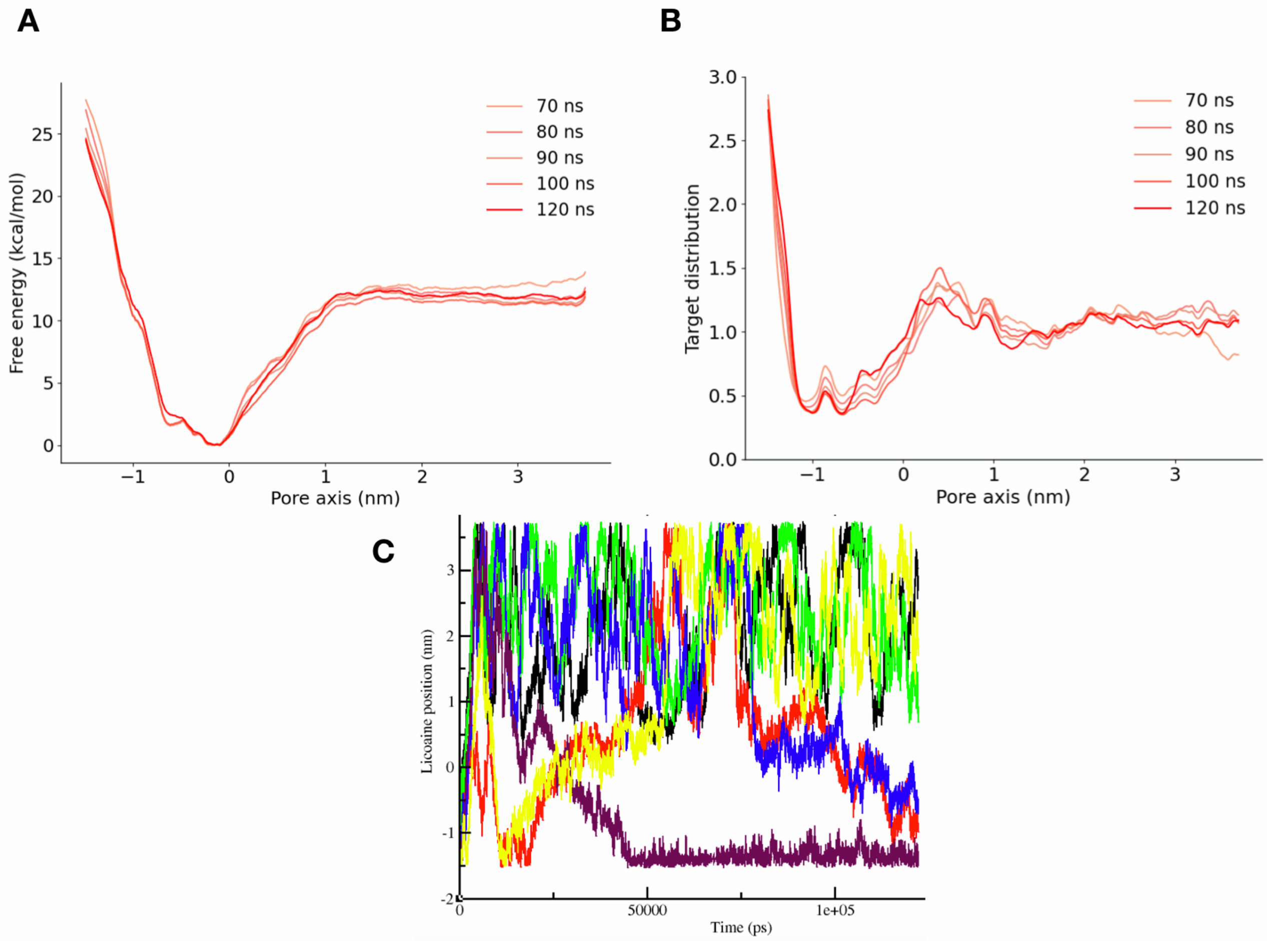

Figure S3: A. Convergence of free energy profile of Lidocaine permeation across a POPC membrane bilayer. **B.** Target distribution at different times. **C.** Evolution of the CV corresponding to six different walkers over time.

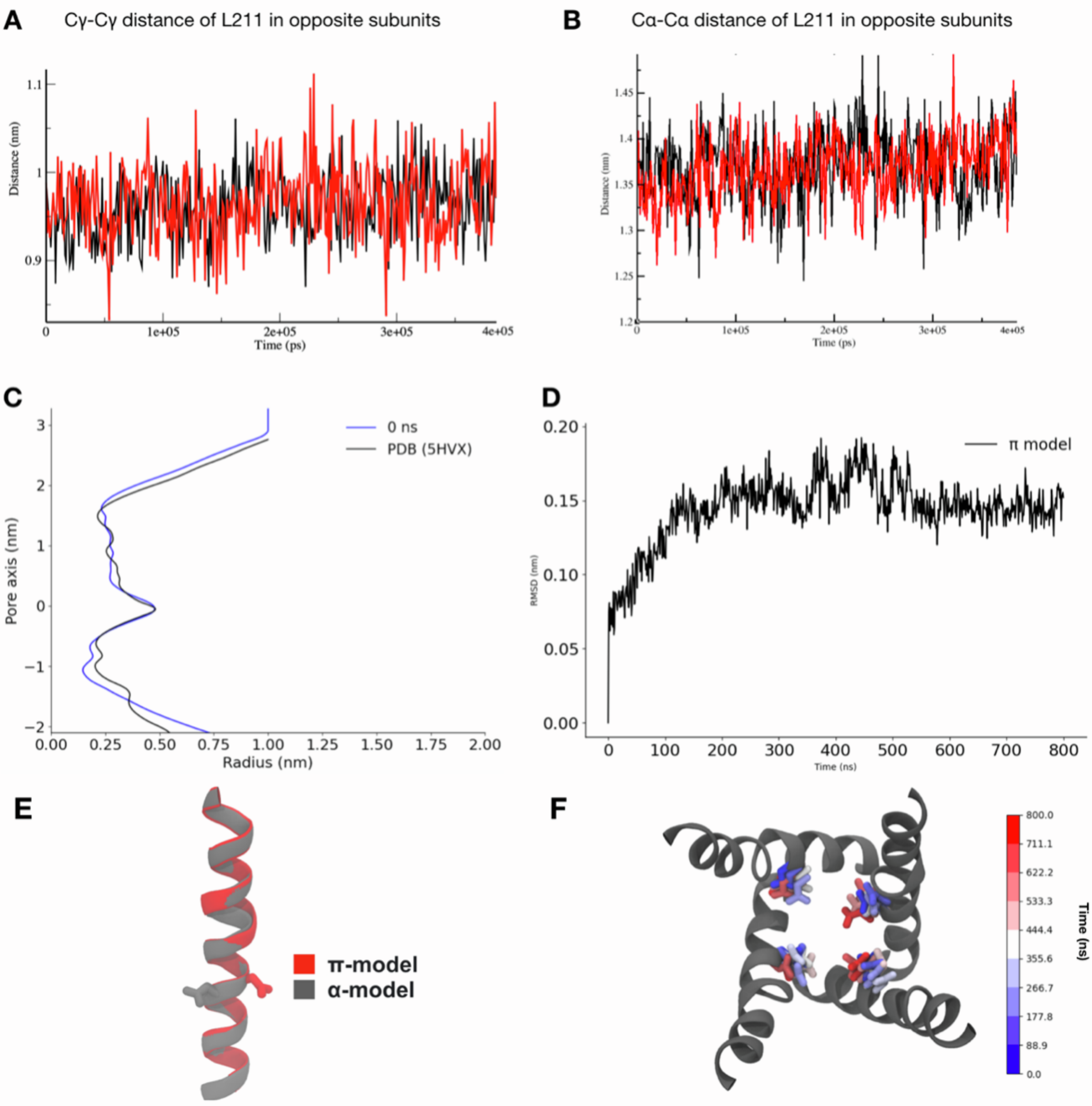

Figure S4. A. Leu-211 opposite subunits Cγ-Cγ distance in the α model. The two distances are shown in black and red. **B.** Leu-211 opposite subunits Cα-Cα distance in the α model. The two distances are shown in black and red. **C.** Pore radius profile of NavMs PDB structure (PDB ID 5HVX - black) and α model after 48 ns of restrained equilibration. **D.** Root mean squared deviation (rmsd) of the backbone atoms of the four S6 helices of the π model, using as reference the first frame of the simulation trajectory. **E.** α model (Grey) superimposed with the π model (Red). **F.** Orientation of Conserved Asparagine in the both the models is shown as sticks.

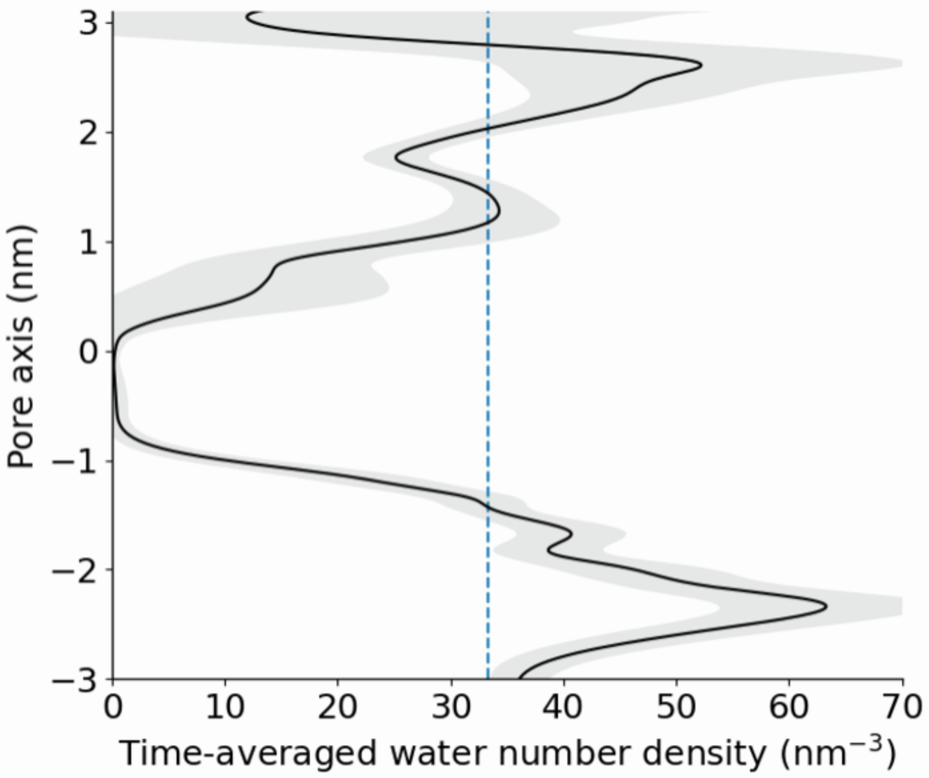

Figure S5: Time averaged water number density of backbone restrained simulation of the α model of NavMs.

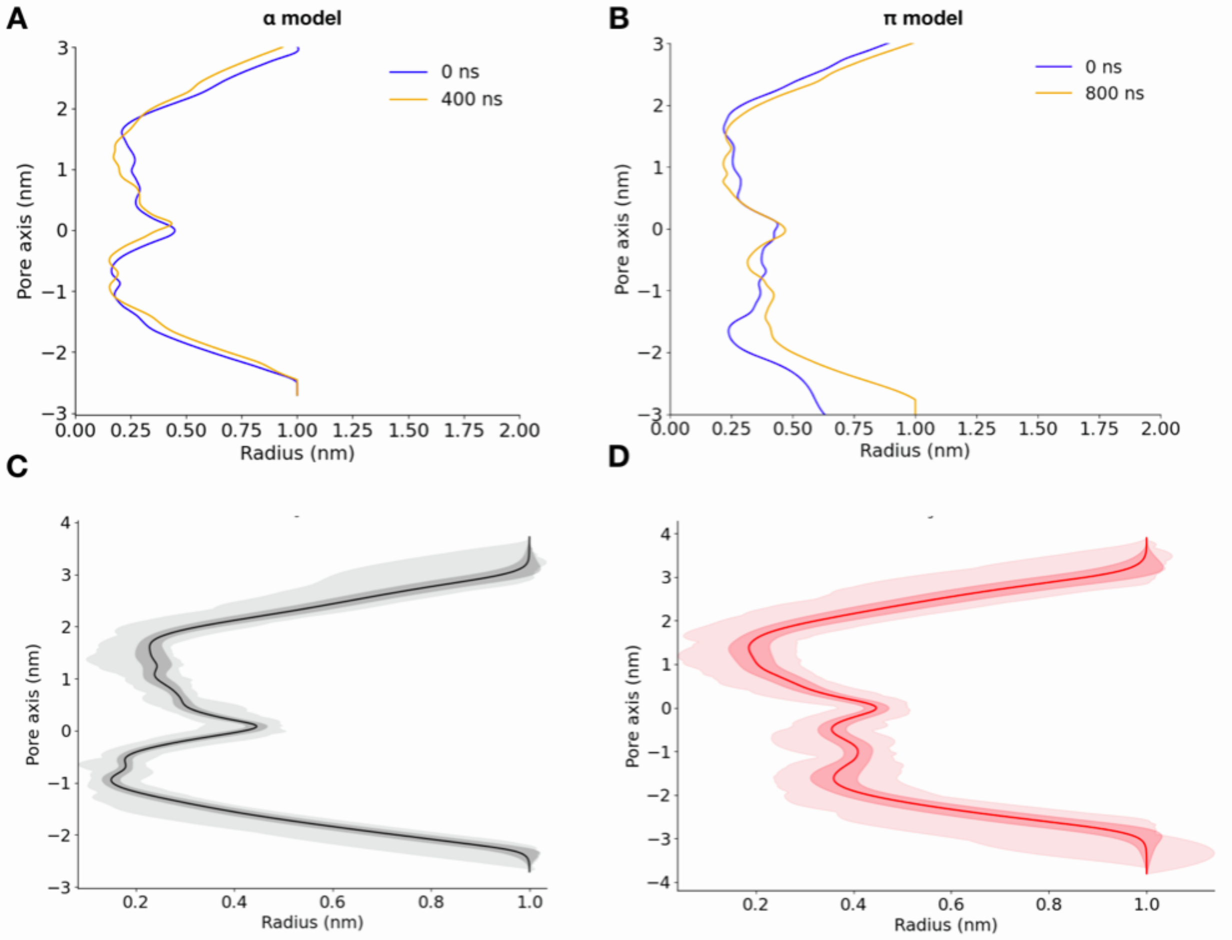

Figure S6: A. Pore radius profile of the α model at the start (0 ns) and end (400 ns) of the equilibrium simulation **B.** Pore radius profile of π model at the start (0 ns) and end (800 ns) of the equilibrium simulation **C.** Time-averaged pore radius profile for the α model **D.** Time-averaged pore radius profile for the π model. Dark shaded region show the standard error on the mean, light shaded region the extreme values.

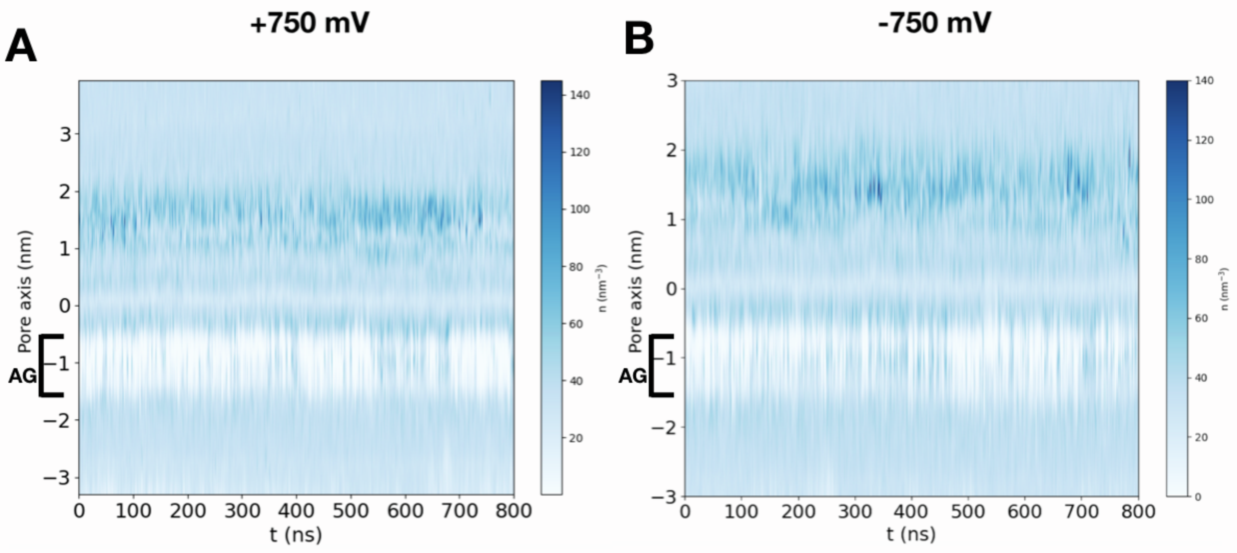

Figure S7: A. Water number density profile over time along the central pore axis at -750 mV. The labelled activation gate (AG) is transiently hydrated **B.** Water number density profile over time along the central pore axis at +750 mV. The labelled activation gate (AG) is transiently hydrated

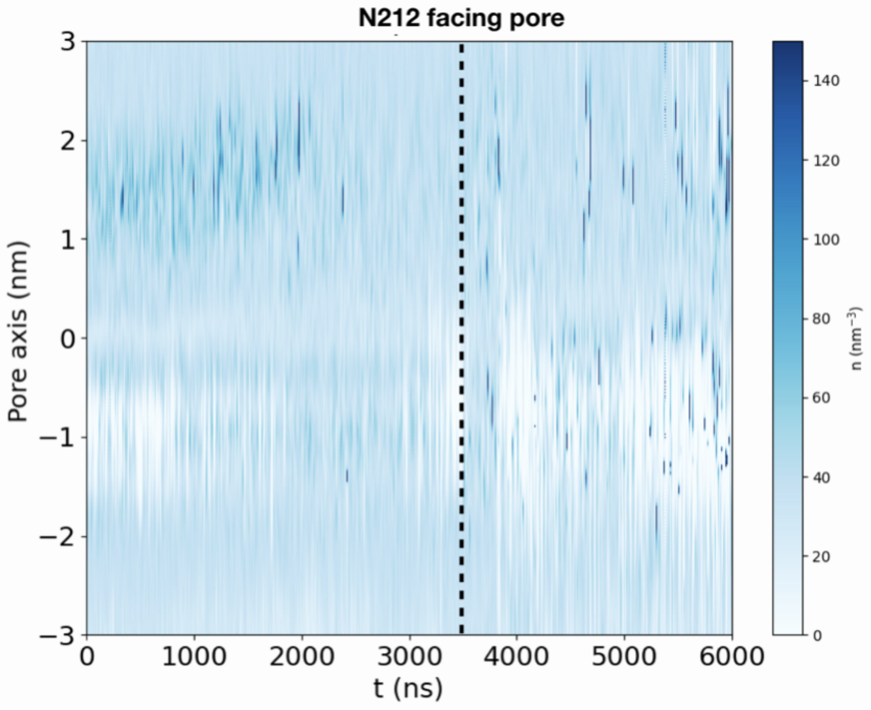

Figure S8: Water number density profile over time along the central pore axis at -750 mV. The activation gate is transiently hydrated, preceding the kinking of one of the S6 helices and subsequent reorientation of the conserved Asn into a pore-facing position (black dotted line).

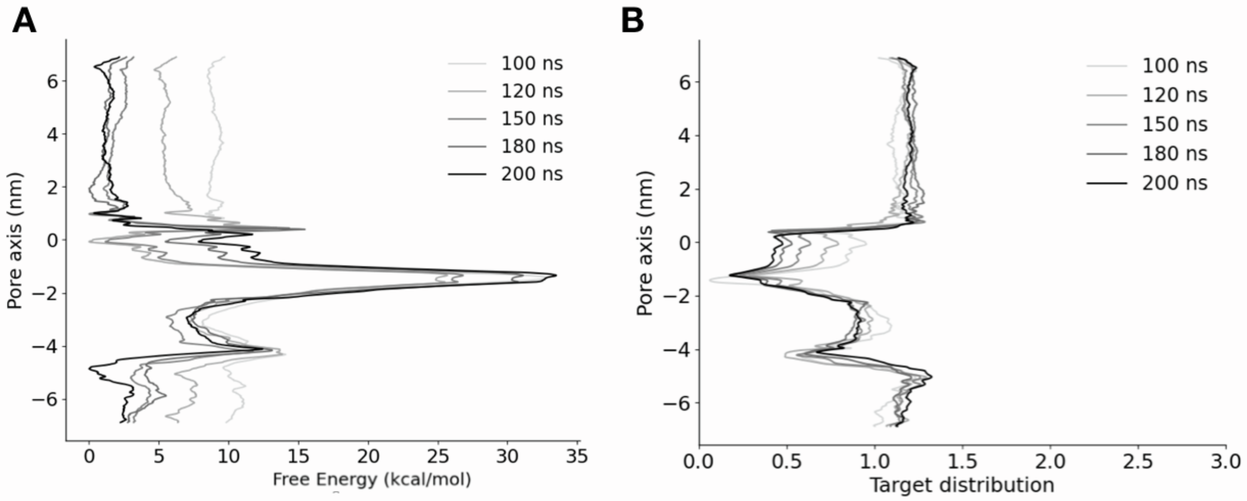

Figure S9: A. Convergence of free energy profile of sodium ion permeation in the NavMs α model. The free energy was calculated across 6 walkers sharing the bias. **B.** Target distribution at different times.

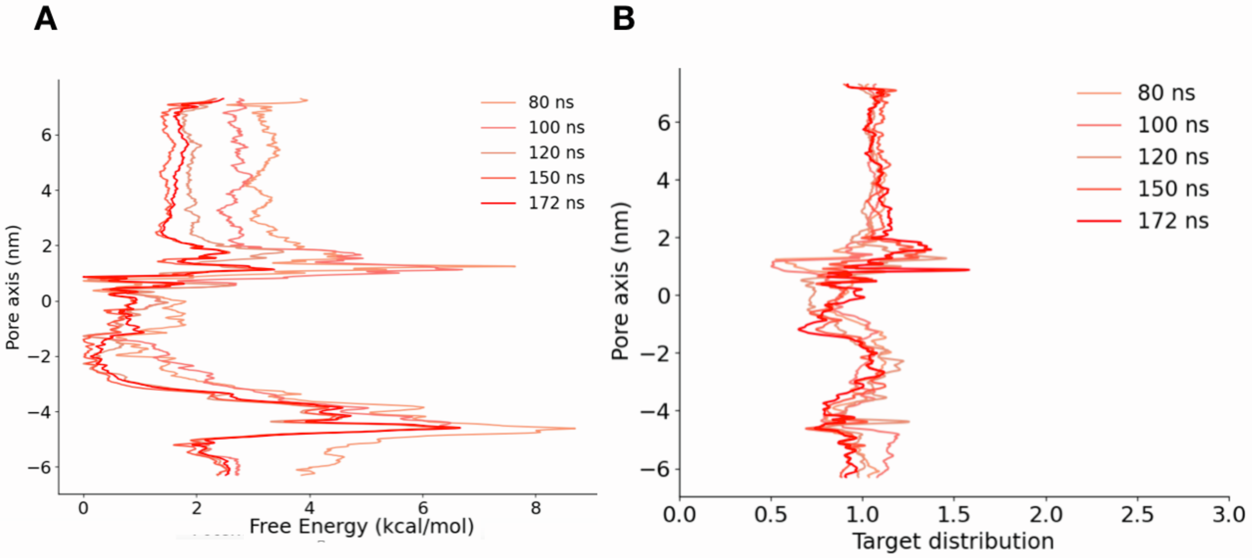

Figure S10: A. Convergence of free energy profile of sodium ion permeation in the NavMs π model. The free energy was calculated across 6 walkers sharing the bias. **B.** Target distribution at different times

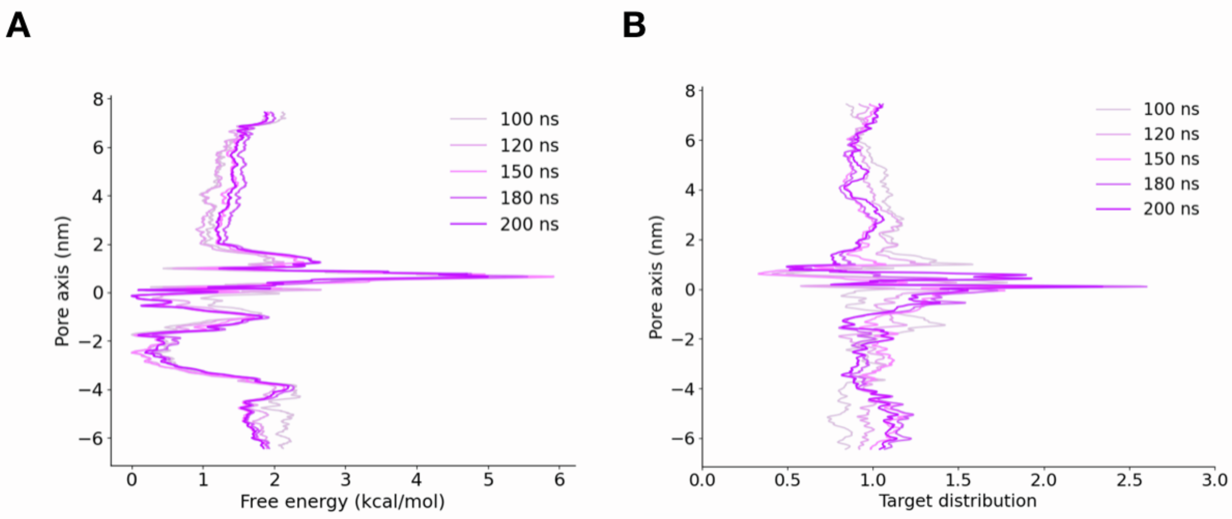

Figure S11: A. Convergence of free energy profile of sodium ion permeation in the NavMs π model mutant N212L. The free energy was calculated across 6 walkers sharing the bias. **B.** Target distribution at different times

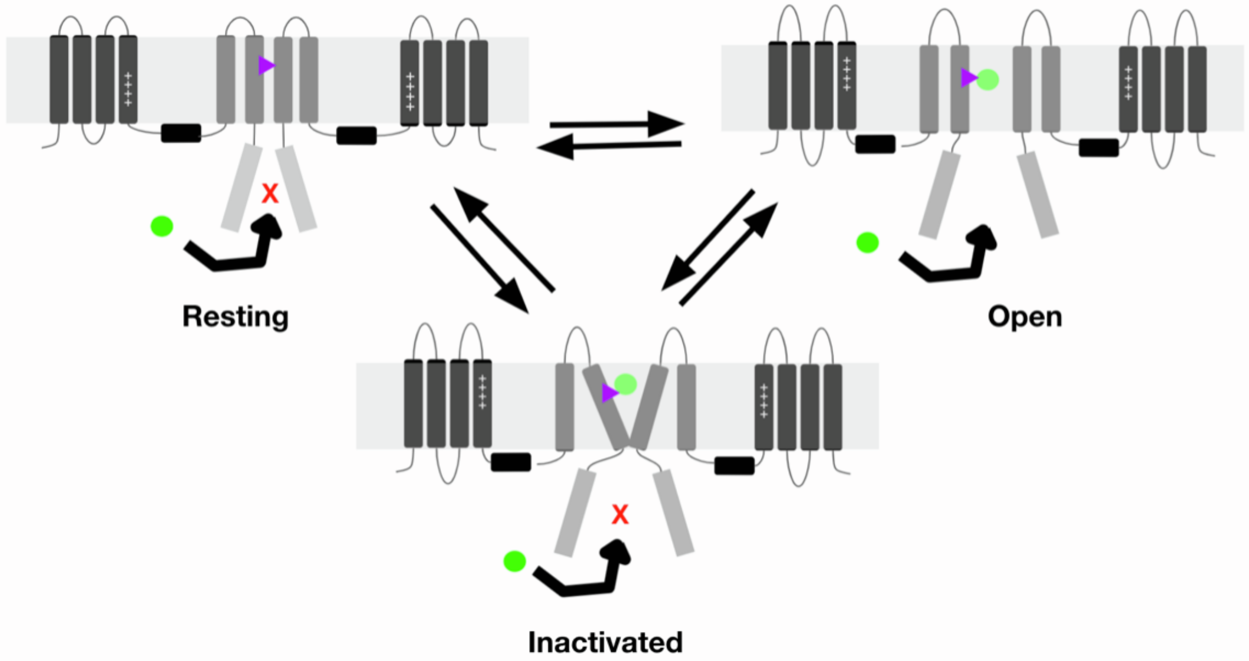

Figure S12: Guarded receptor hypothesis. In the resting and inactivated states, the pore is closed. This prevents the drug (green circle) from accessing its binding site (shown as purple triangle) inside the pore. In the open state, the pore is open which allows the drugs to access its binding site without any hindrance.

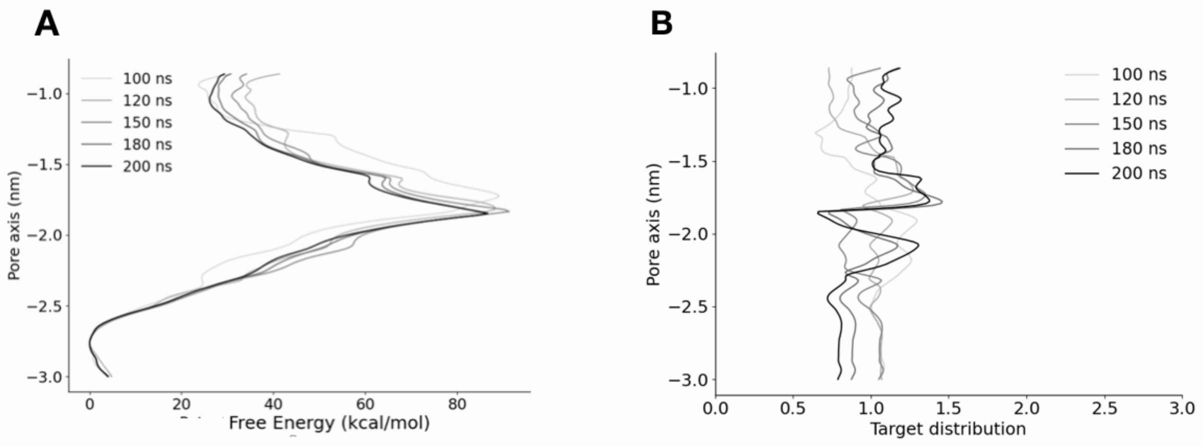

Figure S13: A. Convergence of free energy profile of lidocaine permeation in the NavMs α model. The free energy was calculated across 6 walkers sharing the bias. **B.** Target distribution at different times

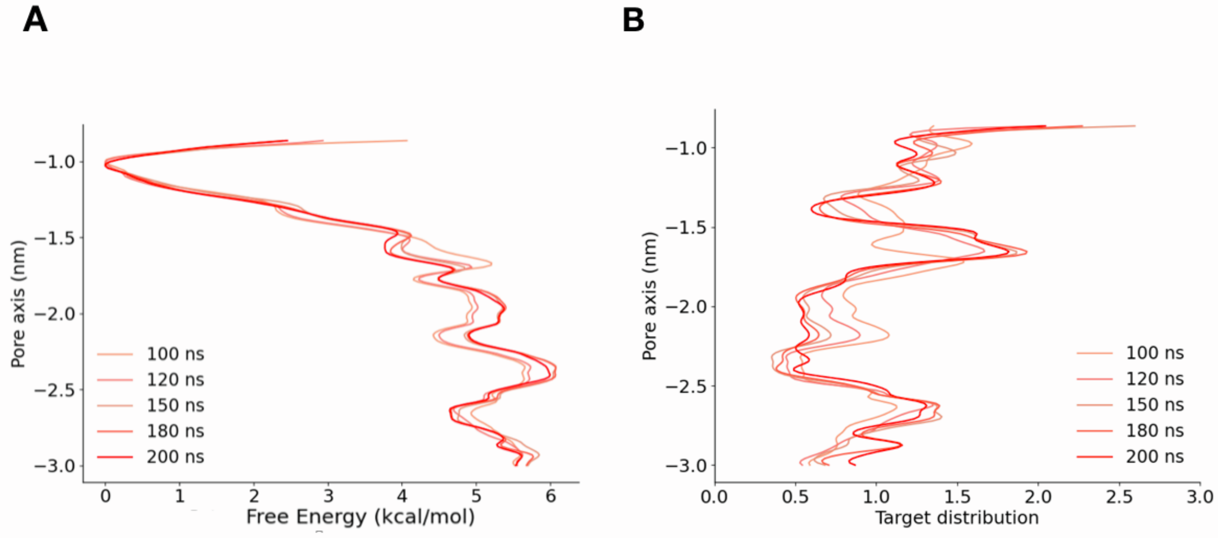

Figure S14: A. Convergence of free energy profile of lidocaine permeation in the NavMs π model. The free energy was calculated across 6 walkers sharing the bias. **B.** Target distribution at different times

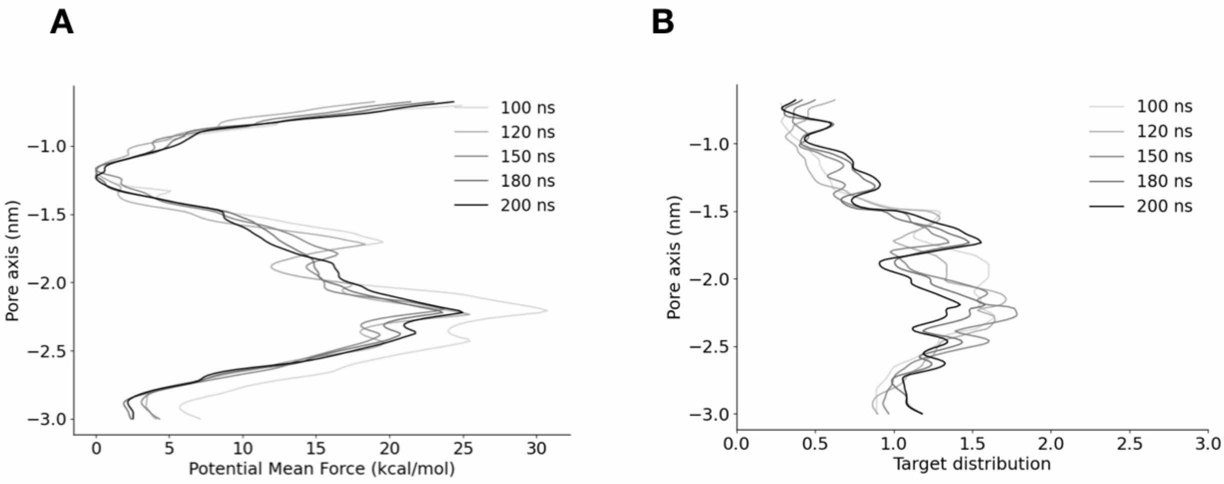

Figure S15: A. Convergence of free energy profile of flecainide permeation in the NavMs α model. The free energy was calculated across 6 walkers sharing the bias. **B.** Target distribution at different times

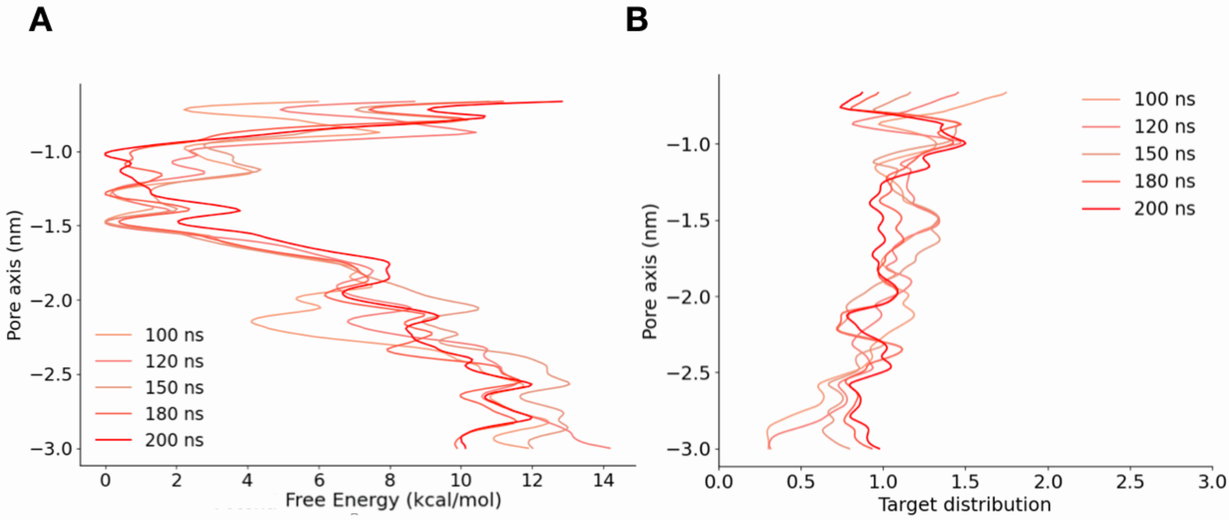

Figure S16: A. Convergence of free energy profile of flecainide permeation in the NavMs π model. The free energy was calculated across 6 walkers sharing the bias. **B.** Target distribution at different times

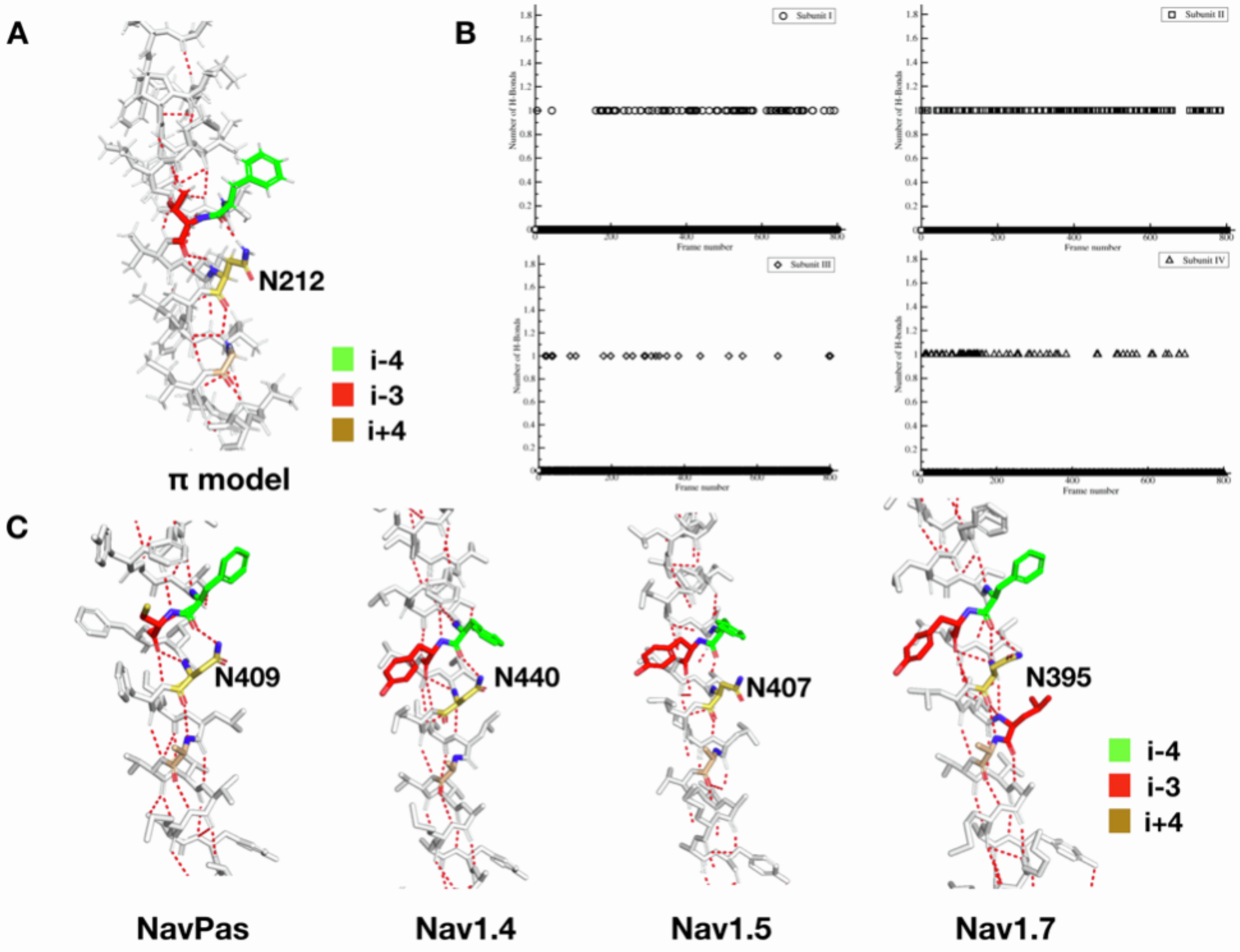

Figure S17:A. A snapshot from the π-model simulation at 200 ns showing a h-bond between the side-chain of conserved Asn-212 (at position i) and the backbone carbonyl of Phe-208 (at position i-4). **B.** H-bond between the side-chain of conserved Asn-212 and the backbone carbonyl of Phe-208 along the 800 ns long simulation of different subunits of the π-model. **C.** H-bond pattern of the conserved Asparagine in the first subunit of different eukaryotic sodium channel. The structures were obtained from the Protein data bankPDB (NavPas PDB ID - 6A91, Nav1.4 PDB ID - 6AGF, Nav1.5 PDB ID - 6UZ3, Nav1.7 PDB ID - 6J8I). The h-bond between the conserved Asparagine residue and the backbone carbonyl of the residue four positions above it is believed to be stabilizing a π-helix.

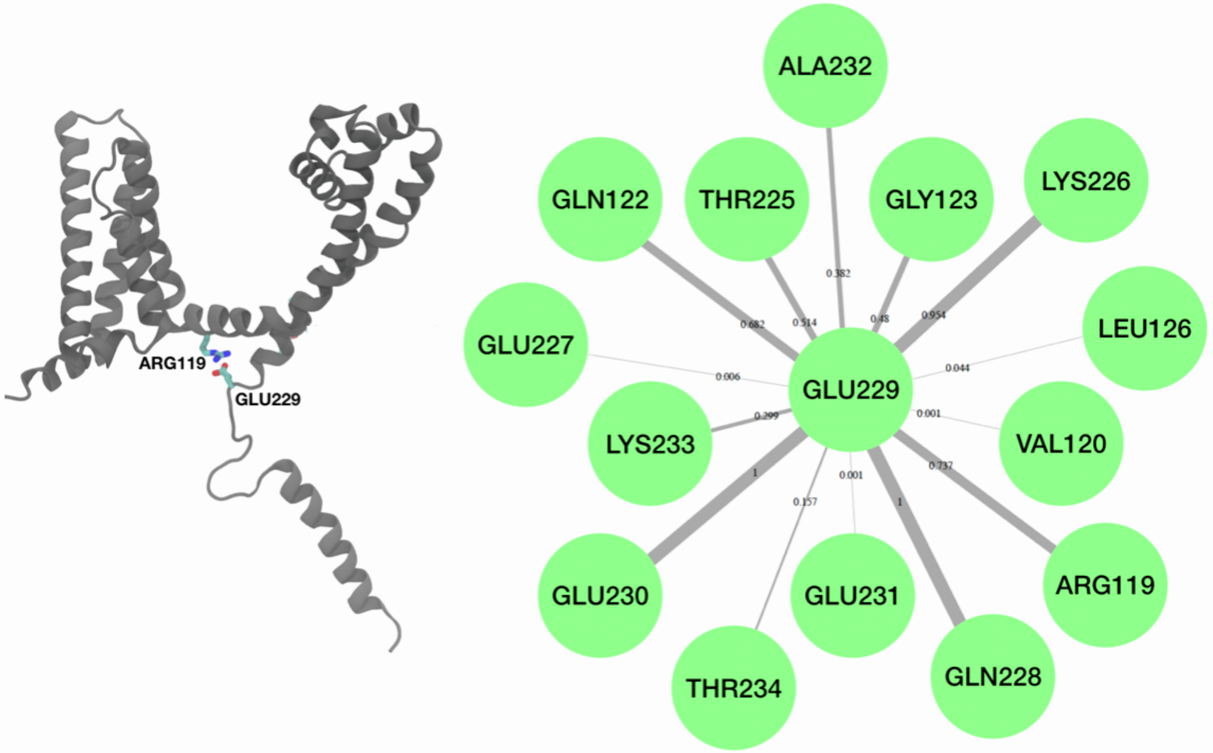

Figure S18: Interaction between R119 and E219, stabilizing an open conformation of the pore in the π-model **B.** Contacts formed by E229 during the 800 ns long simulation of π model, revealing that E229 is in contact with R119 for a significant portion of the time. The thickness of the line is proportional to the fraction of time the contact is formed (black number). Contacts were defined when the distance between the Cβ atoms of pairs of residues was below 6.7 Å , as calculated using MD-TASK (1)

References

1. Brown, D. K., Penkler, D. L., Sheik Amamuddy, O., Ross, C., Atilgan, A. R., Atilgan, C., & Tastan Bishop, Ö. 2017. MD-TASK: a software suite for analyzing molecular dynamics trajectories. *Bioinformatics*, *33:* 2768-2771.
